# A case report of Villar’s nodule in a woman without surgical history

**DOI:** 10.1016/j.ijscr.2018.10.066

**Published:** 2018-11-01

**Authors:** Nabonswindé Lamoussa Marie Ouédraogo, Safy Ilboudo, Abdoul Karim Ouattara, Aimé Sosthène Ouedraogo, Maurice Zida, Nayi Zongo, Dorcas Obiri-Yeboah, Edgard Ouangre, Jacques Simpore, Si Simon Traore

**Affiliations:** a Service of General Surgery of Saint Camille Hospital of Ouagadougou, 01 BP 444 Ouagadougou 01, Burkina Faso; b General and Digestive Surgery Department of Yalgado Ouedraogo University Hospital Center (CHU.YO), Burkina Faso; c Pietro Annigoni Biomolecular Research Center (CERBA)/LABIOGENE, University Ouaga 1 Prof. Joseph KI ZERBO, UFR/SVT. 03BP 7021 Ouagadougou 03, Burkina Faso; d Gynecology Service of Saint Camille Hospital of Ouagadougou, 01 BP 444 Ouagadougou 01, Burkina Faso; e Department of Microbiology and Immunology, School of Medical Sciences, University of Cape Coast, Cape Coast, Ghana

**Keywords:** Endometriosis, Umbilical primitive, Villar’s nodule, Burkina Faso

## Abstract

**Introduction:**

Villar’s nodule is an umbilical endometriosis without anterior or ongoing pelvic endometriosis. The primitive location of this nodule at the umbilical level is rare. Its etiopathogenesis remains unclear.

**Presentation of case:**

We report a case of umbilical endometriosis with unusual clinical expression in a woman in the reproductive years with no surgical history and no known history of endometriosis. Endometriosis manifested as progressive transformation of the normal umbilicus into several small nodules, with bleeding coinciding with the menstrual cycle. The diagnosis was confirmed by histology and surgical treatment consisted of omphalectomy.

**Discussion:**

Umbilical endometriosis is a rare disease that occurs naturally in patients with pelvic endometriosis. Etiopathogenesis of the disease is still unclear. In our patient, the appearance of the nodule was impressively, by a gradual transformation of the normal aspect of the umbilicus, into several small, slightly pigmented, firm, painful and concomitantly bleeding nodules during periods of menstruation. In the literature, this multinodular or budding form would be of exceptional observation.

**Conclusion:**

The characteristics of the umbilical tumor, associated with the cyclical nature of tumor bleeding in a patient without previous history of endometriosis, strongly suggest the diagnosis of Villar’s nodule, but the confirmation is still histological. The treatment is always surgical and recurrence is very rare.

## Highlights

• We report a case of umbilical endometriosis with unusual clinical expression in woman with no know history of endometrios.
• There was a gradual transformation of the umbilicus originally normal appearance into several small nodules.
• The patient underwent mini-omphactomy with uneveventful postoperative course.

## 1 Introduction

Villar’s nodule is a rare phenomenon, first discovered by Mr. Villar in 1886. He then defined it as the presence of endometrial glands in the umbilicus in a woman without a history of pelvic endometriosis [1]. Its frequency is estimated at 0.5–1% of ectopic endometriosis. It occurs in women during the reproductive years. Given the variety of its clinical expression, diagnostic errors are not negligible [2]. It requires adequate care. Through this clinical case, the authors report the clinical, paraclinical and therapeutic aspects of a rare case of Villar’s nodule in the light of a review of the literature. This case report is in line with the SCARE criteria [3].

### 1.1 Presentation of case

**Fig. 1:**
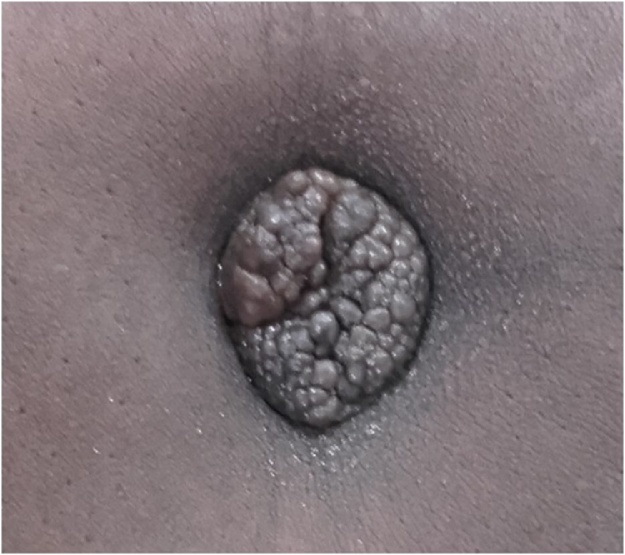
Villar’s nodule, multinodular. Slightly pigmented 2.5 cm long axis.

A 43 years old patient with history of four pregnancies resulting in three parties and a miscarriage, was received in surgery consultation for abdominal pain associated with tender umbilical swelling. The symptoms have evolved about six months with a gradual transformation of the umbilicus originally normal appearance into several small nodules. This swelling flared up during the menstrual cycle, with a recrudescence of pain during these periods. There was no history of metrorrhagia, dysmenorrhea, or dyspareunia. The menstrual cycle was regular. This mass would have motivated a traditional treatment without success. On clinical examination the patient was in good general condition and there were no signs of clinical anemia. There was a multinodular umbilical tumor about 2 cm in size, coarsely pigmented, painful, firm in consistency and letting a bloody liquid flow under pressure. The mass was irreducible, non-pulsating, and not expansive to coughing. Clinical examination reported normal findings. The abdominopelvic Doppler ultrasound had noted a hypoechoic, well-vascularized mass around the navel measuring 2.5 × 2 cm (Fig. 1). The uterus and appendages were normal in appearance.

There were no abnormalities of the abdominal-pelvic contents. The histology of the operative specimen noted macroscopically a piece of 4 × 3 × 3 cm, with the presence of budding nodule partially covered with a skin coating.

**Fig. 2:**
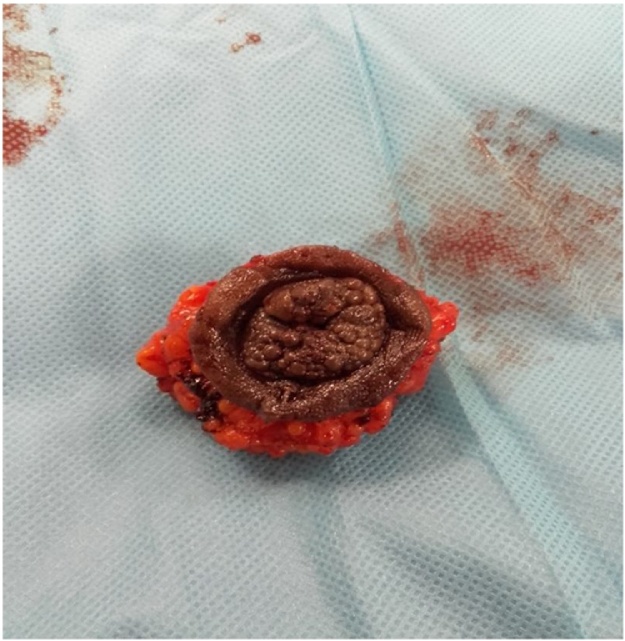
Omphalectomy piece.

At microscopy, it was an epidermal coating without atypia, on fibrous connective tissues, with glandular formations of endometrial type without atypia. These endometrial glands were dilated, distended and hemorrhagic, on a scanty chorion without atypia. The resection margins were normal in appearance. The diagnosis of umbilical endometriosis was made. The patient underwent a mini laparotomy omphalectomy (Fig. 2). The exploration of the abdominal cavity did not identify other localizations of endometriosis. The postoperative course was uneventful with good skin healing in two weeks.

## 2 Discussion

Umbilical endometriosis is a rare disease that occurs naturally in patients with pelvic endometriosis [4]. The Villar’s nodule, also known as primary umbilical endometriosis, is an entity that is very rarely seen in women during the reproductive years with no history of past or present endometriosis. This is the case of our patient. Its frequency is estimated at 0.5–1% of all endometriosis with ectopic localization [2,4]. The average age of onset would be 35–40 years. The observation before the age of 20 would be exceptional [5]. Several theories have tried to explain its etiopathogenesis but until today it remains unclear. The primary umbilical localization could be explained by the direct extension of the endometrial cells through the round ligament or the omphalo-mesenteric canal and by the migration of these cells via the blood and lymphatic vessels of the peritoneal cavity. According to Yu CY et al. [6], the umbilicus being a physiological scar, would be a site of choice for endometrial cells. Usually, the clinical manifestation would be in 40.5% of the cases by the appearance of a painful umbilical nodule of variable aspect, discolored or rather hyperpigmented with bleeding coinciding with the menstrual cycle [1]. In our patient, the appearance of the nodule was by a gradual transformation of the normal aspect of the umbilicus, into several small, slightly pigmented, firm, painful and concomitantly bleeding nodules during periods of menstruation. In the literature, this multinodular or budding form is an exceptional observation [1,7].

**Fig. 3:**
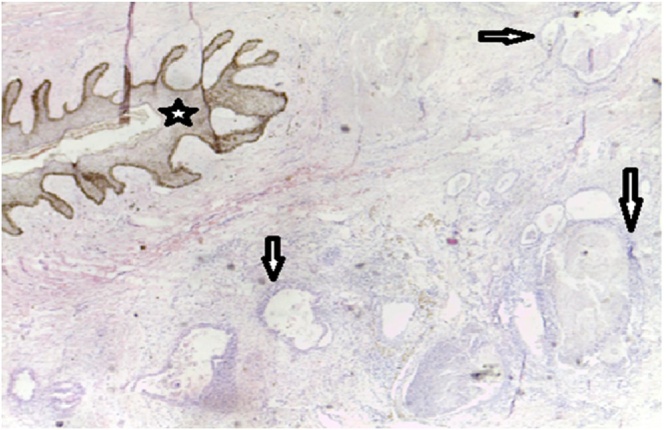
Endometriosis, G40, HE staining. Endometrial glands of variable size sometimes cystic within a cytogenic chorion (arrows) under an epidermis (star) without atypia.

**Fig. 4:**
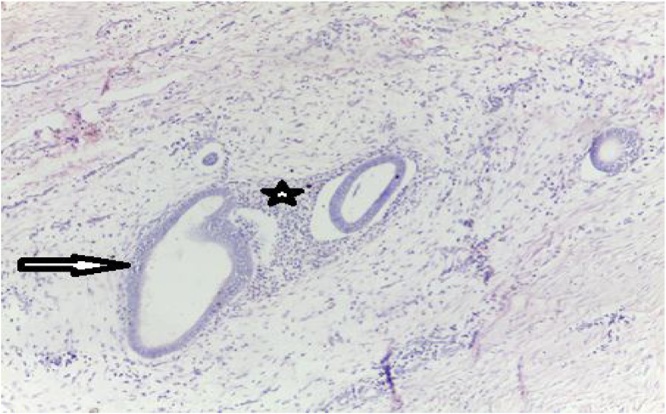
Endometriosis, G100 staining HE Endometrial glands of variable size (arrow) within a cytogenic chorion (star).

The Villar’s nodule in this case can therefore pose a problem of differential diagnosis with the Sister Marie Joseph’s nodule. Indeed, the sister Marie Joseph’s nodule is a skin manifestation in the umbilicus, an intra-abdominal malignancy [8,9]. Magnetic resonance imaging is the preferred morphological examination in this case to evoke the diagnosis of endometriosis. Our patient, however, did not benefit from this examination, but received a Doppler ultrasound which noted a hypoechoic, well vascularized fibromatous mass, developed at the expense of the navel measuring 2.5 × 2 cm, with a normal appearance of uterus and appendices. The clinical diagnosis of umbilical endometriosis was made based on the key element of intermittent bleeding in relation to the menstrual cycle. But the diagnostic confirmation is always made by the histology of the operative specimen [1,2,4,10]. In our patient, the histological examination of the omphalectomy tissue sample confirmed the diagnosis by showing glandular formations of endometrial type without atypia, dilated, distended and hemorrhagic (Fig. 3, Fig. 4). The gold standard of Villar's nodule treatment is surgery that allows complete excision of the tumor [[10], [11], [12], [13]]. It is the treatment of choice realized in our patient who benefited from omphalectomy by mini-laparotomy.

The post-therapeutic evolution is generally favorable with a very low risk of recurrence or very low cancer transformation [1,10,11,14]. Our patient received regular follow-up with consultations every three months, and there were no signs of local recurrence or other localization of endometriosis, with a 12-month follow-up.

### 2.1 Conclusion

The characteristics of the umbilical tumor, associated with the cyclical nature of tumor bleeding in a patient without previous history of endometriosis, strongly suggest the diagnosis of Villar’s nodule, but the confirmation is still histological. The treatment is always surgical and recurrence is very rare.

## Conflict of interest statement

The authors declare that they have no competing interests regarding the publication of this manuscript.

## Sources of funding

No sponsors to declare.

## Ethical approval

Ethical approval is not needed for this case report as patient consent and we are not trialing a new device.

## Consent

We obtained consent to publish this case presentation from the patient.

## Author contribution

**Case report concept and design**: OUEDRAOGO NLM, IBOUDO S, SIMPORE J, TRAORE SS. Acquisition of data: OUEDRAOGO NLM, IBOUDO S. **Statistical analysis and interpretation of data**: OUEDRAOGO NLM, OUATTARA AK, SIMPORE J. **Drafting of the manuscript**: OUEDRAOGO NLM, OUATTARA AK, SIMPORE J. **Critical revision of the manuscript for important intellectual content**: OUEDRAOGO NLM, IBOUDO S, OUATTARA AK, OUEDRAOGO AS, ZIDA M, ZONGO N, OBIRI-YEBOAH D, OUANGRE E, SIMPORE J, TRAORE SS. All authors approved the final version of this publication.

## Registration of research studies

N/A

## Guarantor

Dr Nabonswinde Lamoussa Marie OUEDRAOGO

Prof. Jacques SIMPORE

## Provenance and peer review

Not commissioned, externally peer-reviewed.
